# Clinical features and prognostic factors of spinal fibroblastic/myofibroblastic tumors: a long-term, single-center, retrospective study

**DOI:** 10.7717/peerj.10530

**Published:** 2020-12-16

**Authors:** Haitao Sun, Shaohui He, Yuechao Zhao, Chen Ye, Xinghai Yang, Wei Xu, Jianru Xiao

**Affiliations:** Spinal Tumor Center, Department of Orthopaedic Oncology, Changzheng Hospital, Shanghai, China

**Keywords:** Fibroblastic/myofibroblastic tumor, Spine, En bloc excision, Clinical features, Prognosis, Radiotherapy, Survial analysis, Ki-67, Chemotherapy

## Abstract

**Background:**

Spinal fibroblastic and myofibroblastic tumors (FMTs) are extremely rare. Few studies have reported on the features and outcomes of this condition that affects the axial skeleton. We explored the clinical characteristics and factors affecting the prognosis of spinal FMTs.

**Methods:**

We retroactively assessed the survival of 51 patients with spinal FMTs who underwent surgical and adjuvant treatments in our center between April 2006 and September 2018. Factors affecting disease-free survival (DFS) and overall survival (OS) were analyzed using the Kaplan–Meier method. Variables with *p* value ≤ 0.05 were subjected to multivariate analysis using the Cox proportional hazards regression model. A two-sided *P* value < 0.05 was considered statistically significant.

**Results:**

The mean follow-up period was 50.8 ± 35.6 months (Range 4.2–172.6). Kaplan–Meier survival curves showed that the 5-year DFS was 10% (95% CI [31.09-42.56]) and the 5-year OS was 53% (95% CI [61.28–97.20]). Multivariate analysis showed that en bloc excision was associated with better DFS (HR 0.214, 0.011) and OS (HR 0.273, 0.043), radiotherapy negatively affected OS (HR 0.353, 0.033), and the recurrence and Ki-67 index <5% significantly affected DFS (HR 3.008, 0.008 and 2.754, 0.029).

**Conclusions:**

Spinal FMTs are rare. Surgery is the treatment of choice and en bloc excision is strongly recommended to improve outcomes. Disease recurrence and the Ki-67 marker are correlated with the progression of these tumors.

## Introduction

Fibroblastic and myofibroblastic tumors (FMTs) are relatively common soft tissue neoplasms that range from reactive to neoplastic (Alaggio et al., 2010; Altomare et al., 2010). FMTs are a type of soft tissue tumor classified by the WHO as having three sub-groups, including benign or reactive tumors, malignant tumors, and intermediate tumors, which are defined as locally aggressive and potentially metastasizing neoplasms(Amendola et al., 2014; Baglole et al., 2006).

FMTs may arise in multiple anatomic locations throughout the body in variable proportions (Bhowmick, Neilson & Moses, 2004; Boriani, Weinstein & Biagini, 1997; Ceballos et al., 2000). However, bony involvement by the tumors is less frequent (Chugh et al., 2010; Coffin & Alaggio, 2012; Desmoulière, Guyot & Gabbiani, 2004; Eyden, 2000; Eyden, 2001; Eyden, 2005). FMTs of the spine are rare, regardless of infringement of the vertebral column or appendices. Chronic back pain and numbness are the most common complaints associated with these tumors. Additional symptoms, ranging from dyskinesia to sphincter disturbances, are also reported secondary to spinal FMTs. Surgical intervention is commonly recommended to remove the oncological lesions, relieve spinal compression, and recover nerve function.

The clinical features and outcomes of spinal FMTs have been sporadically reported (Eyden et al., 2009; Ferrari et al., 2013; Fletcher, 1998). The prognostic factors affecting spinal FMTs must be clarified. We reported the clinical features of spinal FMTs and investigated the prognostic factors affecting disease-free survival (DFS) and overall survival (OS) by reviewing eligible patients with spinal FMTs.

## Materials & Methods

### Data collection

Fifty-one patients with spinal FMTs received consecutive surgical treatments and adjuvant therapies at our center between April 2006 and September 2018. We collected demographic and relevant clinical data of the patients from their medical records in a retrospective review. The data included baseline information, clinical symptoms, blood biochemical indices, and tumor locations and involved the scope, pathological classification, and details of the therapeutic regimen. The Changzheng Hospital Ethics Committee approved our study protocol (No. D3108N0525), and written informed consent was obtained from all patients or their legal guardians.

Contrast-enhanced computed tomography (CT) and magnetic resonance imaging (MRI) with gadolinium enhancement were used to detect bony destruction and soft tissue involvement. The Eastern Cooperative Oncology Group (ECOG) scoring system and Frankel score were used to evaluate the preoperative performance and neurological status, respectively. The individualized surgical protocol, including en bloc resection (marginal or wide excision) and piecemeal excision, was designed for all the patients according to the Tomita classification (Tomita et al., 2001) and Weinstein-Boriani-Biagini (WBB) surgical staging system (Boriani, Weinstein & Biagini, 1997). Radiotherapy and chemotherapy were administered based on the surgical outcome and tumor pathology. The use of bisphosphonate was introduced to the patients with osteolytic lesions. Histopathology and immunochemical staining were reviewed postoperatively. The scoring of Ki-67 staining depended on the definite immunochemical results, and was expressed as a percentage of positivity. Other immunochemical indicators were provided in the form of positivity or negativity.

### Follow-up strategy

The follow-up period started from the date of surgery and ended on December 20th, 2018. DFS was the primary endpoint in our study and OS was the secondary endpoint. DFS was defined as the duration from the date of surgery to the date of the first evidence of recurrence/metastasis on the basis of the clinical manifestations and imaging changes, death caused by relevant diseases, or the end of follow-up care. OS was defined as the duration from the date of surgery to death or the end of follow-up care. Follow-up care was routinely performed either by outpatient visits or telephone interviews every 3 months for the first 6 months, every 6 months for the next 2 years, and then annually until care was discontinued.

### Statistical analysis

Quantitative data are described by mean/median (standard deviation, SD), and qualitative data are reported by counts (percentages, 95% confidential interval [CI]). The chi-square test or Fisher exact method was used for categorical data, and student *t*-test was used for quantitative data when appropriate.

Univariate and multivariate analyses were performed to investigate the factors affecting DFS and OS for patients with spinal FMTs. The log-rank test was used in univariate analysis, and variables with a *p* < 0.05 in a univariate analysis were subjected to the Cox proportional hazards regression model to identify independent prognostic factors. DFS and OS rates were estimated using the Kaplan–Meier method. A two-sided *p* < 0.05 were considered statistically significant. All statistical analyses were conducted using SPSS statistics, version 21.0 (IBM, Armonk, New York).

## Results

### Demographic data

A total of 51 patients (24 male and 27 female) with FMTs in the axial skeleton were surgically treated with adjuvant therapies at our institution. Detailed patient information is found in Table 1.

The patients ranged in age from 15 to 70 years with a mean of 31.6 ± 16.1 years, including 28 (54.9%) patients younger than 25 years of age. The most common symptom was chronic endurable pain with or without radiating pain. Attendant symptoms included numbness of the extremities, progressive dyskinesia, segmental limitation of motion, varying degrees of sphincter disturbances, and weight loss. Weight loss was defined as a > 5% reduction in body weight (BW) within 6 months. The duration of preoperative symptoms ranged from 0.25 to 144 months (9.0 ± 21.2 m).

Thirty-eight (74.5%) patients had previously received treatments in other hospitals and were referred to our hospital because of recurrence and the remaining 13 patients were initially identified with primary FMTs in our hospital. The preoperative neurologic and performance status of the patients was assessed using the Frankel score 19 and ECOG 20, respectively.

**Table 1:** General information of patients with FBT/MFBTs.


Age (y)(mean ± SD, range)	31.6 ± 16.1,15–70
Duration of symptom (mo) (mean ± SD, range)	9.1 ± 21.2, 0.25–144
Symptoms	
Chronic pain and radiating pain (±)	31(60.8%)
Numbness of extremities	29(56.9%)
Progressive dyskinesia	19(37.2%)
Segmental movement restriction	2(3.9%)
Sphincter disturbances	6(11.8%)
Weight loss	21(41.2%)
Preoperative NS (Frankel score 19)	
B	1(2.0%)
C	6(11.8%)
D	25(49.0%)
E	19(37.3%)
Preoperative PS(ECOG 20)	
0	1(2.0%)
1	33(64.7%)
2	12(23.5%)
3	3(2.9%)
4	2(3.9%)
Tumor location	
Cervical portion	15(29.4%)
Thoracic portion	15(29.4%)
Lumbar portion	8(15.7%)
Sacrococcygeal portion	13(25.5%)
No. of involved segment	
1	8 (15.7%)
2	7 (13.7%)
3	20 (39.2%)
≥3	16 (31.4%)
Pathology	
Benign (%)	5(9.8%)
Local Aggressiveness (%)	9 (17.6%)
Sporadic Metastatic (%)	26 (51.0%)
Malignancy (%)	11(21.6%)
Surgical Strategy	
En bloc Fashion	
Marginal (%)	10(19.6%)
Wide (%)	5(9.8%)
Piecemeal (%)	36(70.5%)
Operation Time (m)(mean ± SD, range)	264.5 ± 128.9, 80-570
Intraoperative Blood Loss (ml)(mean ± SD, range)	992.3 ± 847.2, 200-4000
Follow-up Time (mo) (mean ± SD, range)	50.8 ± 35.6, 4-173
Disease-Free Survival	
2-year survival (%)	75.6%
5-year survival (%)	12.7%
Overall Survival	
2-year survival (%)	95.8%
5-year survival (%)	57.3%

**Notes.**

Abbreviation NS Neurological Status PS Performance Status

The tumor location and involved segments were recorded for further analysis.

Solitary lesions were detected in 47 cases (92.2%) and multiple lesions were detected in four cases (7.8%). The lesions were evaluated by the WBB and Tomita classification systems. The vertebral body was affected in 18 cases (35.3%) (sector 5-9 or 4-8 by WBB system, sector 1 by Tomita classification), and included involvement of the posterior aspect in 16 cases (31.4%) (sector 3-10 by WBB system, sector 2 or 3 by Tomita classification). Deep vertebral involvement was detected in more than 34 cases (66.7%) (layer C by WBB system and I-III by Tomita classification). However, only three lesions (5.9%) were defined in the vertebrae (layer B-C by WBB system). There were 19 confirmed cases in which the tumor extended into the epidural space (39.6%) (layer D by WBB system and IV by Tomita classification). Soft tissue involvement was observed in 40 cases (83.3%).

### Radiographical findings

**Figure 1:**
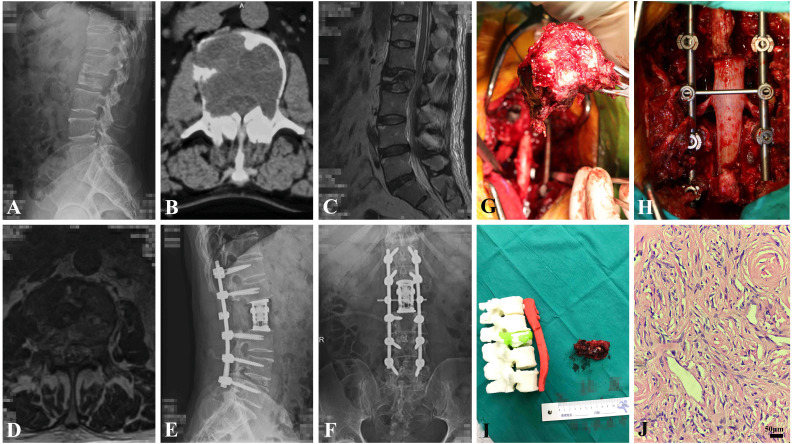
The clinical data of a 61-year-old male patient (adult fibrosarcoma). (A) Preoperative oblique conventional radiograph of lumbar segment. (B) Axial CT image of L2 level reveals bony erosion. (C) T2-weighted sagittal MRI image of L2 shows pathological fracture. (D) T2-weighted axial MRI image of L2 shows. (E and F) Postoperative A-P and lateral X-ray show the instrumentation. (G) Gross pathological specimen after en bloc resection of L2 vertebral body and tumor. (H) intraoperative photography showing reconstruction of lumbar segment. (I) The 1:1 3D printing model of lumbar segment and the gross specimen. (J) Photomicroscopy of tumor shows pathological mitotic activity, evident focal cell atypia and proliferation of spindle-shaped cells of fibrohiscytic sarcomatosis (Hematoxylin and Eosin stain, x 400).

CT scan findings included a heterogeneously or homogeneously asymmetric mass with adjacent vertebral marginal osteogenic changes, and intense but inhomogeneous enhancement following the administration of contrast agent. Some lesions appeared as radiolucent, shuttle-shaped expansile osteolysis with thinning of the bone cortex. MRI T1-weighted imaging showed hypo- to iso-intensity signals, with T2-weighted imaging hyperintensity or mixed intensity. A peripheral enhancement effect was occasionally observed after the administration of contrast agent (Figs. 1C, 1D).

### Treatment and outcomes

The SINS (spinal instability neoplastic score) system was used to evaluate the spinal stability and assist with the surgical protocol. This score was less than seven in 10 cases (21.4%), 7–12 in 29 cases (63.0%), and more than 12 in 7 cases (15.2%). Fifteen patients (29.4%) were treated surgically by en bloc method (marginal and wide), and 36 tumors were removed piecemeal because of the large tumor size, adjacent neurovascular structure, and/or multiple vertebral extensions. Fourteen patients (27.4%) underwent surgical removal using a combined anterior and posterior approach, while vertebrectomies and sagittal resections were applied to tumor removal through a single posterior or anterior approach in 37 patients (72.5%). The representative patient who underwent en bloc resection is depicted in Figs. 1G–1I.

Thirty-six tumors (70.5%) were removed in a piecemeal fashion. 15 patients (29.4%) with an appropriate tumor location received en bloc surgical treatment. Three patients experienced a local recurrence after a marginal resection, and metastasis occurred in one patient after a wide resection. Of the patients receiving a piecemeal resection, seven patients (13.7%) underwent extra postoperative radiotherapy, while chemotherapy was administered to 15 patients (29.4%). The chemotherapy regimen was determined by the pathological subtype of spinal FMTs. Nine patients (15.7%) with inflammatory myofibroblastic tumors received ALK tyrosine kinase inhibitors. The sarcoma-like regimen of isosfamide (12–15 mg/m^2^) and doxorubicin (75 mg/m^2^) was used in 10 patients (adult fibrosarcoma, low grade fibromyxoid sarcoma, low grade myofibroblastic sarcoma). Chemotherapy produced varied results in spinal FMTs. Six patients with inflammatory myofibroblastic tumors developed local recurrence after the administration of ALK tyrosine kinase inhibitors. Follow-up imaging showed relapse in three patients with adult fibrosarcoma, one patient with low grade fibromyxoid sarcoma, and two patients with low grade myofibroblastic sarcoma after chemotherapy. The chemotherapy regimen was restarted until the next surgery or tumor regression. Zoledronic acid was used to prevent osteolytic bone destruction in 23 patients (45.1%). Bone-related events were not observed in patients with the use of bisphosphonate.

### Pathology

The histologic diagnoses were as follows: fibromatosis colli (two cases), desmoplastic fibroblastoma (five cases), calcifying fibrous tumor (one case), desmoid-type fibromatoses (three cases), lipofibromatosis (three cases), solitary fibrous tumor (12 cases), low grade myofibroblastic sarcoma (four cases), inflammatory myofibroblastic tumor (14 cases), hyalinizing spindle cell tumor (one case), low grade fibromyxoid sarcoma (two cases), and adult fibrosarcoma (four cases). The histological images are shown in Fig. 1J.

**Figure 2:**
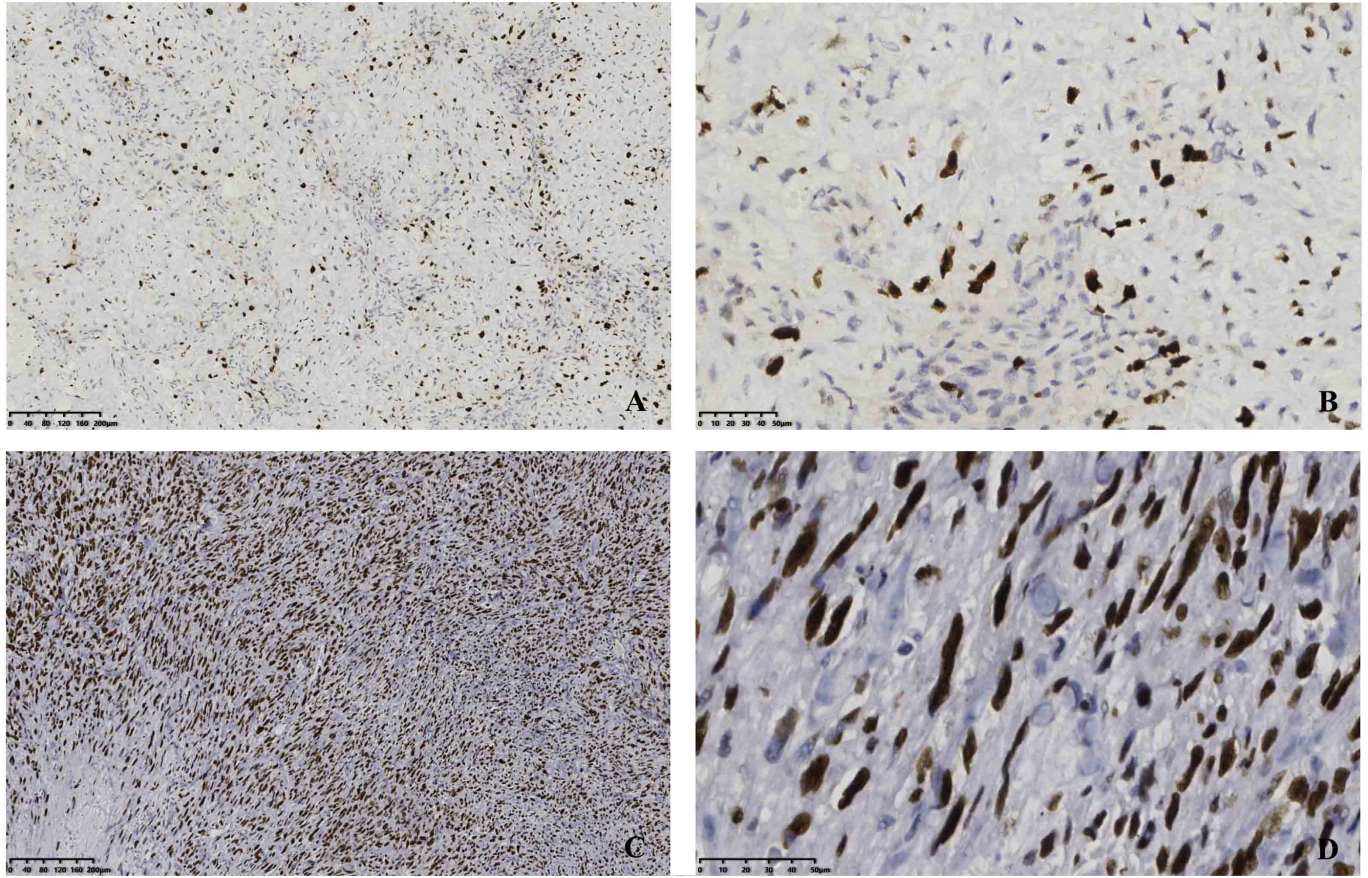
Immunostaining of a resection specimen with primary antibodies to Ki-67 shows diffusely positive cells. (A–B) Immunopositivity for Ki-67 with low proliferation index; (C–D) Immunopositivity for Ki-67 with high proliferation index. Original magnification ×100(A&C), original magnification ×400(B&D).

In our study, the positive immunohistochemistry showed the following: vimentin (12/14, 85.7%), SMA (33/36, 91.7%), CD34 (7/11, 63.6%), Bcl-2 (14/18, 77.8%), *β*-catenin (12/18, 66.7%), CK (5/10, 50%), ALK (+) (10/14, 71.4%), and EMA (1/12, 8.3%), respectively. The Ki-67 range was tested in all cases (9.71 ±  7.81%, 9%). Our immunochemical findings revealed that the tumor cells were positive for the different scoring of Ki-67 (Fig. 2).

### Complications and recurrence

Wound complications and urinary tract infections were observed in two patients who recovered fully after intensive treatment and meticulous nursing care. One patient (1.9%) suffered incisional disunion, and debridement was subsequently performed. Another patient (1.9%) developed a urinary tract infection on the 5th postoperative day and was treated with oral antibiotics and irrigation of the urinary tract.

During the mean follow-up period of 46.2 months, 25 patients (49.0%) were still alive without any evidence of recurrence or metastasis. All of the surviving patients recovered well postoperatively and had no neurological deficit. Fourteen patients died of tumor-related disease within a mean period of 53.6 ±  20.0 months (25.6–81.7 m). One patient died of surgery-related causes and eleven patients died of other diseases within a mean interval of 61.3 ±  48.1 m (19.7 m-154.6 m, 39.8 m).

Twenty-eight of the 38 patients (74.5%) patients treated at other hospitals developed local recurrences, within a mean follow-up of 30.0 months (11.2–60.8), and 2 patients developed metastasis within an average period of 20.4 months (17.5–23.2). The other 8 recurring cases were patients with a primary tumor who received initial treatment at our institute (seven for piecemeal resection, one for en bloc resection). Two patients developed distant metastasis 30.2 and 39.7 months after surgery, respectively.

### Prognostic analysis of DFS and OS

The results of univariate analysis of the prognostic factors affecting both DFS and OS are listed in Table 2. As the table shows, patients with age ≤25 (*p* = 0.03), initial admission (*p* = 0.003), Ki-67 ≥5% (*p* = 0.007), multicentricity (*p* = 0.015), metastatic pathology (*p* = 0.008) and piecemeal surgical treatment (*p* = 0.001) had lower DFS. Tumor location (*p* = 0.014), surgical resection style (*p* = 0.013), recurrence on admission (*p* = 0.04), weight loss (*p* = 0.04), and combined radiotherapy (*p* = 0.029) were unfavorably associated with OS.

**Figure 3:**
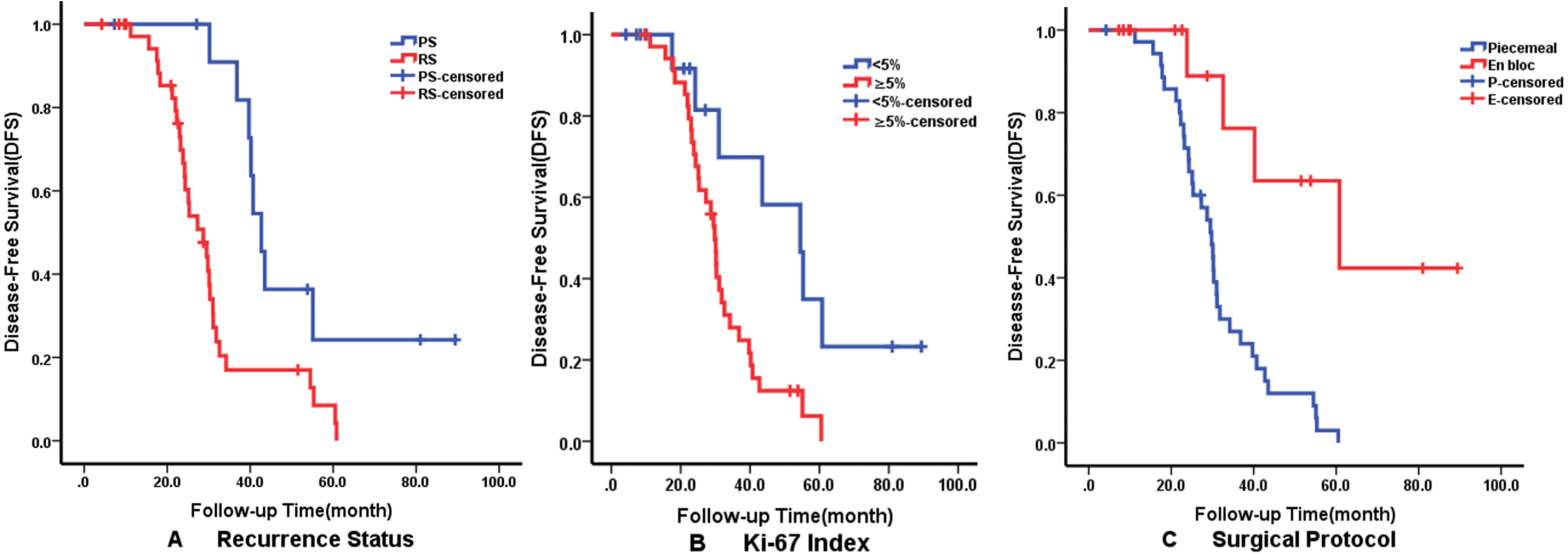
Kaplan–Meier curves showing disease-free survival (DFS) relating to different factors. Kaplan–Meier curves of disease-free survival (DFS) for (A) Recurrence Status; (B) Ki-67 Index; (C) Surgical Protocol.

**Figure 4:**
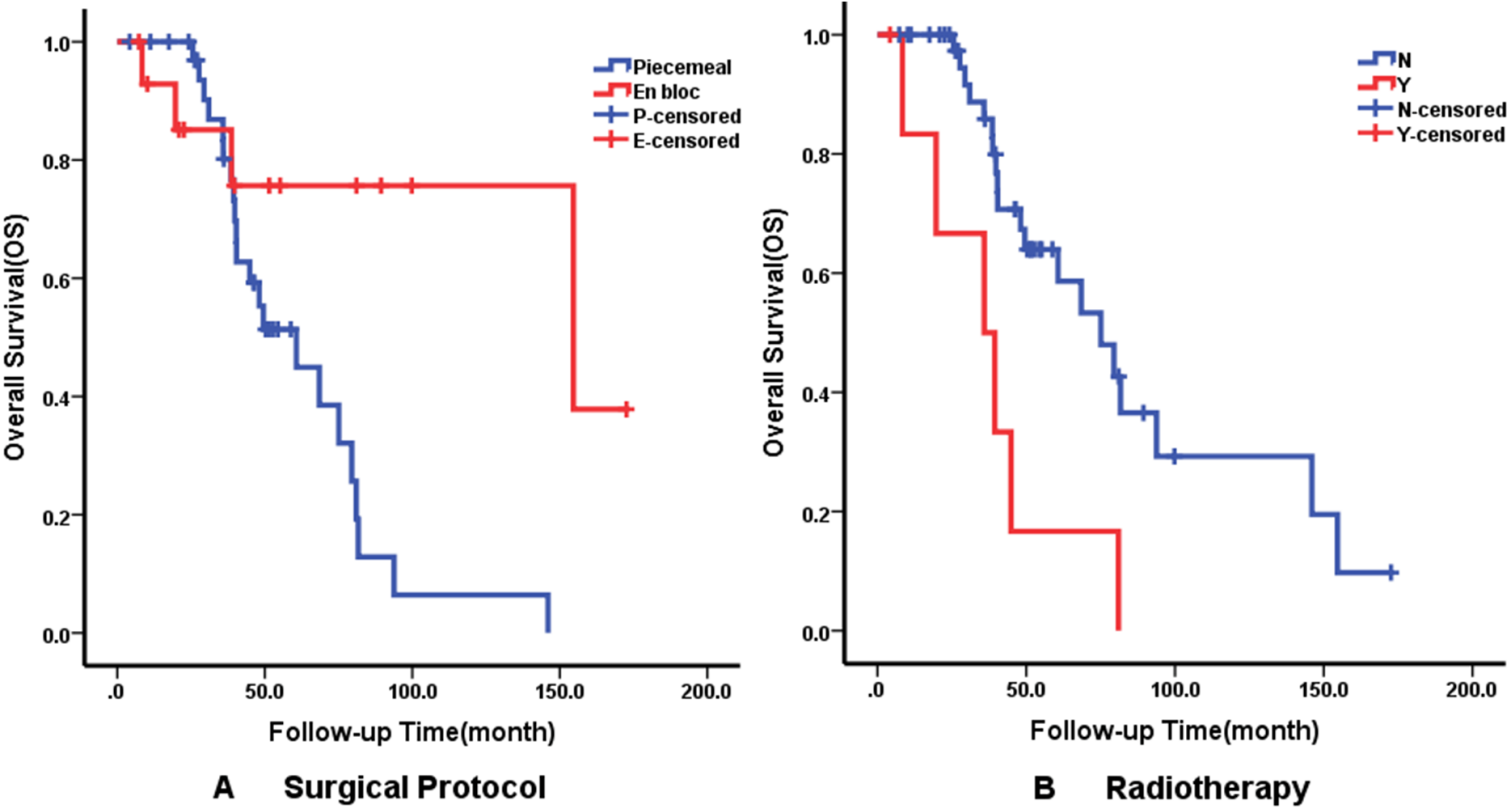
Kaplan–Meier curves showing overall survival (OS) relating to different factors. Kaplan–Meier curves of overall survival (OS) for (A) Surgical Protocol; (B) Radiotherapy.

Multivariate analysis using the Cox proportional hazards regression model identified that recurrence on admission (*p* = 0.008), Ki-67 ≥5% (*p* = 0.029) and surgical protocol (*p* = 0.011) were independent predictors of DFS. En bloc resection (*p* = 0.020) was correlated with better OS but radiotherapy affected OS negatively. The details of independent prognostic factors are illustrated in Table 3. The Kaplan–Meier survival curves of DFS and OS for their independent predictors are shown in Figs. 3 and 4. The 5-year DFS and OS were 10% and 53%, respectively.

## Discussion

Spinal FMTs are rare but are one of the most common mesenchymal spindle cell lesions of the axial skeleton (Eyden et al., 2009; Laffan, Ngan & Navarro, 2009). However, the clinical features, outcomes and prognostic factors of spinal FMTs are poorly understood. The clinical features and factors affecting prognosis of spinal FMTs based on the data collected in our center.

The classic symptom of FMTs is chronic pain with or without radiating symptoms, which could be transiently relieved by acupuncture, massage, or nonsteroidal anti-inflammatory drugs. Early diagnosis and detection are usually difficult because of the non-specific symptomology. A palpable mass is the second most common symptom. However, the slow growth of the soft tissue mass may decrease the awareness of the disease and multiple spinal segments are often involved at the initial diagnosis.

**Table 2:** Univariate analysis of the prognostic factors affecting disease-free survival and overall survival.

Factor	N	DFS		OS	
		*N*	P	*N*	P
Patient factors				
Age: ≤25/>25	28/23	25/13	0.030^*^	17/9	0.343
Gender: Male/Female	24/27	18/20	0.197	14/12	0.773
Recurrence on admission: N/Y	13/38	78/30	0.003^*^	9/16	0.040^*^
Ki-67 index:<5%/ ≥ 5%	16/35	7/31	0.007^*^	4/22	0.113
Preoperative Frankel: D-E/A-C	29/22	25/13	0.090	16/10	0.674
Preoperative EOCG score:0-2/3-4	46/5	36/2	0.307	24/2	0.944
Duration of symptom(m): ≤1/1-3/ ≥ 3	12∕15∕24	8∕7∕23	0.519	4∕9∕13	0.938
Local pain of spine: N/Y	20/31	11/27	0.578	9/17	0.177
Radiation pain: N/Y	28/23	18/20	0.333	12/14	0.323
Numbness: N/Y	22/29	15/23	0.710	10/16	0.419
Dyskinesia: N/Y	32/19	24/14	0.723	18/8	0.726
Sphincter disturbances: N/Y	45/6	34/4	0.683	23/3	0.117
Loss of weight: N/Y	30/21	21/17	0.285	9/17	0.040^*^
Preoperative SINS: <7/7-12/>12	10∕29∕7	5∕24∕4	0.420	4∕12∕6	0.428
Preoperative Hb(g/L): ≤120/>120	16/29	10/24	0.617	11/12	0.143
preoperative D-D level( *μ*g/mL): ≤200 />200	15/27	12/20	0.307	8/14	0.996
Preoperative LDH(U/L): ≤200/>200	26/7	19/6	0.130	13/4	0.669
Preoperative ESR(mm/h): ≤20/>20	31/9	23/7	0.512	20/2	0.312
Preoperative AGR: ≤1.5/>1.5	17/25	12/20	0.377	8/13	0.996
Preoperative NLR: ≤2.7/.2.7	29/15	24/10	0.869	18/5	0.700
Preoperative NMR: ≤10.0/>10.0	21/23	15/19	0.876	8/15	0.587
Preoperative LMR: ≤5.0/>5.0	24/20	15/19	0.717	9/14	0.914
Tumor factors				
WBB section: anterior(4-9)/posterior(1-3,10-12)/both	18∕16∕14	16∕10∕10	0.441	6∕9∕9	0.393
WBB layers: layer A-B/layer C-D	16/32	11/24	0.911	8/16	0.727
Tumor location: C/T/L/S	15∕15∕8∕13	14∕12∕5∕7	0.084	11∕6∕5∕4	0.014^*^
No. of involved segment:<3/ ≥ 3	23/28	10/28	0.015^*^	6/20	0.188
Multicentricity: N/Y	47/4	35/3	0.613	25/1	0.546
Tomitta classification: A/B	6/42	4/31	0.634	2/22	0.957
Pathology: benign+ local aggressiveness/ sporadic metastasis + malignancy	37/14	34/4	0.008^*^	21/5	0.442
Treatment factors				
Surgical protocol: piecemeal/en bloc excision	36/15	34/4	0.001^*^	22/4	0.013^*^
Surgical approach: anterior/posterior/ both	9∕28∕14	7∕19∕12	0.799	5∕15∕6	0.401
Intraoperative blood loss(ml): ≤1000/>1000	29/20	21/15	0.487	14/11	0.862
Operation time(h): ≤3/3-6/ ≥ 6	16∕20∕15	12∕12∕14	0.070	8∕11∕7	0.308
Bisphosphonate: N/Y	26/25	22/16	0.324	11/15	0.738
Chemotherapy: N/Y	36/15	26/12	0.641	17/9	0.818
Radiotherapy: N/Y	44/7	34/4	0.213	20/6	0.003^*^

**Notes.**

Abbreviations DFS disease-free survival SINS spinal instability neoplastic score LDH lactic dehydrogenase ESR erythrocyte sedimentation rate AGR albumin/globulin ratio NLR neutrophil/lymphocyte ratio NMR neutrophil/monocyte ratio LMR lymphocyte/monocyte ratio

* *P* value less than 0.05 for the univariate analysis.

Overall, spinal FMTs are non-specific on MRI images. Enhancement with gadolinium can effectively define the extension of the lesion (Navarro, 2009). The lesions showed complex intensity in a T1- and T2-weighted image, but gadolinium enhancement was seen clearly in our study. CT is essential to assess the spinal involvement and bony destruction of the spine for further surgical determination. The combined findings of CT and MRI can provide more comprehensive information for diagnosis and therapy.

**Table 3:** Multivariate analysis of prognostic factors affecting DFS and OS.

Factor	DFS		OS	
	HR (95% CI)	P	HR (95% CI)	P
Recurrence: N/Y	3.008(1.326–6.824)	**0.008**	–	0.260
Ki-67 index:<5%/ ≥ 5%	2.754(1.107–6.855)	**0.029**	–	–
Surgical strategy: piecemeal/enbloc(marginal/wide) excision	0.214 (0.065–0.707)	**0.011**	0.273(0.078–0.960)	**0.043**
Radiotherapy: (-)/(+)	–	–	0.353(0.135–0.920)	**0.033**

**Notes.**

CI confidence interval HR hazard ratio

FMTs can be comprehensively identified by their salient features, including the spindled or stellate morphology in histological sections and variable immunochemical reactivity for vimentin, smooth muscle actin (SMA), desmin and calponin (Ceballos et al., 2000; Eyden, 2001; Eyden et al., 2009; Skalli et al., 1988). In cases where interpretation becomes difficult, detection of *α*-SMA immunopositivity in mesenchymal cells other than myofibroblast (Eyden, 2000; Eyden, 2005), and the presence of protomyofibroblasts that do not contain *α*-SMA may help confirm the diagnosis (Eyden et al., 2009). In cases where there is focal or equivocal staining, electron microscopy would be useful when an ambiguous tumor is found to have definitively convincing fibroblastic and/or myofibroblastic features (Fletcher, 1998). We recommend the combined use of electron microscopy and immunohistochemistry to provide precise pathological classification.

Although patients with spinal FMTs present subtle histologic differences, the clinical behaviors may be poles apart, making the diagnosis challenging. Based on the WHO classification, intermediate and malignant spinal FMTs with metastatic potential are an important group that is different from other non-metastatic tumors with cytogenetic or molecular genetic abnormalities (Coffin & Alaggio, 2012). Understanding the different features of FMTs is important for prognostic prediction. Our univariate and multivariate analysis showed that patients with metastatic spinal FMTs had worse DFS. Although there was no significant difference in OS between the intermediate and malignant spinal FMT groups, OS was relatively higher in the non-metastatic group. The number of myofibroblasts and myofibroblastic differentiations are closely associated with the prognosis. The clinical and experimental research has provided evidence that the number of myofibroblasts in tumor stroma is a tumor promoter (Kellermann et al., 2007; Surowiak et al., 2007), while growth factors such as TGF-β secreted from myofibroblasts promote cell proliferation and proteolytic enzymes that degrade the matrix (Baglole et al., 2006; Bhowmick, Neilson & Moses, 2004; Desmoulière, Guyot & Gabbiani, 2004). Myofibroblasts also promote angiogenesis (Orimo & Weinberg, 2006). The Ki-67 labeling index could be relevant to detecting tumor multiplication, knowing that a mesenchymal neoplasm with a high degree of Ki-67 tended to proliferate more rapidly (Zhang et al., 2018; Zhou et al., 2017). We found that the Ki-67 label index was the hallmark of cell proliferation. Both univariate analysis and multivariate analysis confirmed that the Ki-67 labeling index was negatively correlated with DFS. Although survival analysis failed to confirm the effect of Ki-67 on OS, our results still showed that the patients with a low Ki-67 labeling index had obviously higher OS compared with their counterparts. The spinal FMTs with metastatic potential and a high degree of proliferation tend to have a poorer prognosis.

Surgery remains the treatment of choice (Ferrari et al., 2013; Shindle et al., 2002; Zhao et al., 2016) and the surgical quality was often the most significant prognostic factor (Orbach et al., 2010). It is important to emphasize that, for the subtype of FMTs such as desmoid-type fibromatoses, high relapse rates are still observed regardless of surgical resection with histologically free margins (Gronchi et al., 2003; Smith et al., 2000). En bloc excision with marginal free resection is still the best surgical protocol for spinal FMTs. Amendola et al. (Amendola et al., 2014) asserted that en bloc resection is an effective procedure for primitive spinal tumors and can provide a better outcome at the expense of functional limitations. However, surgeons should note that recurrent status, combined anterior and posterior approaches, and multi-segmental excision are significant prognostic factors. Our study indicated that repeated tumor violation and double contemporary approaches increased the risk of contamination of the normal tissue. Although there is no statistical significance among surgical approaches, patients treated with the single surgical approach had lower risk of recurrence.

Surgeons should weigh the overall balance between the complication rate and the success rate in achieving negative margins, knowing that the associated complications might be more difficult to be managed than the tumor itself (Amendola et al., 2014). Tumor contamination of the surgical field should not be neglected in any case of piecemeal excision.

The surgical management of spinal FMTs is a great challenge because of the anatomical complexity of the spine and the involvement of peripheral nerve roots. A wait-and-see policy and less-frequent systemic therapy are the options of multimodality therapy. Systemic chemotherapy has been used with varied results in FMTs (Alaggio et al., 2010; Orbach et al., 2010). The effectiveness of chemotherapy in neo-adjuvant setting had been accepted, but the postoperative value remains to be established (Gabel et al., 2015; Haas et al., 1997; Rodrigues et al., 2017). Conservative management with observation according to tumor presentation has proven effective for subgroups of spinal FMTs (Salas et al., 2011). We recommend no chemotherapy but close surveillance even though there was no statistic discrepancy between the two groups in our study. Radiotherapy has been the alternative or complementary treatment of choice for patients without the option of total gross excision or recurrence. Research has shown that post radiation sarcoma was a rare potential late sequel of ionizing radiation (Korampalli, Mathew & Stafford, 2013). In our study, the long-term follow-up results indicated that radiotherapy might be associated with late adverse events. The validity of hormonal therapy, anti-inflammatory therapy, steroid treatment, or target therapy has been identified in some subgroups of spinal FMTs, but the systemic therapeutic effect for remaining types of spinal FMTs needs further research (Altomare et al., 2010; Chugh et al., 2010).

## Conclusions

Spinal FMTs are relatively rare. Surgery remains the treatment of choice, and en bloc excision is strongly recommend for spinal FMTs to improve the outcome. The recurrence status and a high Ki-67 marker are correlated with worse prognosis. Although this is the first large case series research reporting the clinical features and independent prognostic factors for spinal FMTs, there are some inevitable limitations. The number of patients enrolled in this study makes it difficult to conduct statistical analysis, especially cox regression analysis, and the enrolled patients all received surgery without comparison with none-surgery patients. This was a retrospective study, which is naturally limiting. More multicenter studies should be undertaken to confirm our findings and conclusions.

## Acknowledgements

We thank Professor Xiao Jianru for supporting our study and providing conscientious guidance. The authors also thank our colleagues for their kind help.

##  Supplemental Information

10.7717/peerj.10530/supp-1 Supplemental Information 1 Data and annotation.

## Additional Information and Declarations

### Data Availability

The following information was supplied regarding data availability: Raw data are available as a Supplemental File.

## References

[ref-1] Alaggio R, Cecchetto G, Bisogno G, Gambini C, Calabro ML, Inserra A, Boldrini R, De Salvo GL, ES GdA, Dall’igna P (2010). Inflammatory myofibroblastic tumors in childhood: a report from the Italian Cooperative Group studies. Cancer.

[ref-2] Altomare DF, Rotelli MT, Rinaldi M, Bocale D, Lippolis C, Lobascio P, Cavallini A (2010). Potential role of the steroid receptor pattern in the response of inoperable intra-abdominal desmoid to toremifene after failure of tamoxifen therapy. International Journal of Colorectal Disease.

[ref-3] Amendola L, Cappuccio M, De Iure F, Bandiera S, Gasbarrini A, Boriani S (2014). En bloc resections for primary spinal tumors in 20 years of experience: effectiveness and safety. Spine Journal.

[ref-4] Baglole CJ, Ray DM, Bernstein SH, Feldon SE, Smith TJ, Sime PJ, Phipps RP (2006). More than structural cells, fibroblasts create and orchestrate the tumor microenvironment. Immunological Investigations.

[ref-5] Bhowmick NA, Neilson EG, Moses HL (2004). Stromal fibroblasts in cancer initiation and progression. Nature.

[ref-6] Boriani S, Weinstein JN, Biagini R (1997). Primary bone tumors of the spine. Terminology and surgical staging. Spine (Phila Pa 1976).

[ref-7] Ceballos KM, Nielsen GP, Selig MK, O’Connell JX (2000). Is anti-h-caldesmon useful for distinguishing smooth muscle and myofibroblastic tumors? An immunohistochemical study. American Journal of Clinical Pathology.

[ref-8] Chugh R, Wathen JK, Patel SR, Maki RG, Meyers PA, Schuetze SM, Priebat DA, Thomas DG, Jacobson JA, Samuels BL, Benjamin RS, Baker LH (2010). Efficacy of imatinib in aggressive fibromatosis: results of a phase II multicenter Sarcoma Alliance for Research through Collaboration (SARC) trial. Clinical Cancer Research.

[ref-9] Coffin CM, Alaggio R (2012). Fibroblastic and myofibroblastic tumors in children and adolescents. Pediatric and Developmental Pathology.

[ref-10] Desmoulière A, Guyot C, Gabbiani G (2004). The stroma reaction myofibroblast: a key player in the control of tumor cell behavior. International Journal of Developmental Biology.

[ref-11] Eyden B (2000). Smooth-muscle-type myofilaments and actin in reactive and neoplastic nonmuscle cells. Ultrastructural Pathology.

[ref-12] Eyden B (2001). The myofibroblast: an assessment of controversial issues and a definition useful in diagnosis and research. Ultrastructural Pathology.

[ref-13] Eyden B (2005). The myofibroblast: a study of normal, reactive and neoplastic tissues, with an emphasis on ultrastructure. part 2—tumours and tumour-like lesions. Journal of Submicroscopic Cytology and Pathology.

[ref-14] Eyden B, Banerjee SS, Shenjere P, Fisher C (2009). The myofibroblast and its tumours. Journal of Clinical Pathology.

[ref-15] Ferrari A, Alaggio R, Meazza C, Chiaravalli S, Vajna de Pava M, Casanova M, Cavaliere E, Bisogno G (2013). Fibroblastic tumors of intermediate malignancy in childhood. Expert Review of Anticancer Therapy.

[ref-16] Fletcher CD (1998). Myofibroblastic tumours: an update. Verhandlungen der Deutschen Gesellschaft für Pathologie.

[ref-17] Gabel BC, Goolsby M, Hansen L, HS U (2015). Inflammatory myofibroblastic tumor of the left sphenoid and cavernous sinus successfully treated with partial resection and high dose radiotherapy: case report and review of the literature. Cureus.

[ref-18] Gronchi A, Casali PG, Mariani L, Lo Vullo S, Colecchia M, Lozza L, Bertulli R, Fiore M, Olmi P, Santinami M, Rosai J (2003). Quality of surgery and outcome in extra-abdominal aggressive fibromatosis: a series of patients surgically treated at a single institution. Journal of Clinical Oncology.

[ref-19] Haas RL, Keus RB, Loftus BM, Rutgers EJ, Van Coevorden F, Bartelink H (1997). The role of radiotherapy in the local management of dermatofibrosarcoma protuberans. Soft Tissue Tumours Working Group. European Journal of Cancer.

[ref-20] Kellermann MG, Sobral LM, Da Silva SD, Zecchin KG, Graner E, Lopes MA, Nishimoto I, Kowalski LP, Coletta RD (2007). Myofibroblasts in the stroma of oral squamous cell carcinoma are associated with poor prognosis. Histopathology.

[ref-21] Korampalli TS, Mathew B, Stafford ND (2013). Post radiation myofibrosarcoma of hypopharynx. Journal of Surgical Case Reports.

[ref-22] Laffan EE, Ngan BY, Navarro OM (2009). Pediatric soft-tissue tumors and pseudotumors: MR imaging features with pathologic correlation: part 2. Tumors of fibroblastic/myofibroblastic, so-called fibrohistiocytic, muscular, lymphomatous, neurogenic, hair matrix, and uncertain origin. Radiographics.

[ref-23] Navarro OM (2009). Imaging of benign pediatric soft tissue tumors. Seminars in Musculoskeletal Radiology.

[ref-24] Orbach D, Rey A, Cecchetto G, Oberlin O, Casanova M, Thebaud E, Scopinaro M, Bisogno G, Carli M, Ferrari A (2010). Infantile fibrosarcoma: management based on the European experience. Journal of Clinical Oncology.

[ref-25] Orimo A, Weinberg RA (2006). Stromal fibroblasts in cancer: a novel tumor-promoting cell type. Cell Cycle.

[ref-26] Rodrigues C, Cabral D, Almodovar T, Ribeiro A, Delgado D, Mota L, Mendes S, Alvoeiro M, Torres C, Calado T, Antunes M, Felix F (2017). Unusual behavior of a lung inflammatory myofibroblastic tumor: case report. Revista Portuguesa de Cirurgia Cardio-Torácica e Vascular.

[ref-27] Salas S, Dufresne A, Bui B, Blay JY, Terrier P, Ranchere-Vince D, Bonvalot S, Stoeckle E, Guillou L, Le Cesne A, Oberlin O, Brouste V, Coindre JM (2011). Prognostic factors influencing progression-free survival determined from a series of sporadic desmoid tumors: a wait-and-see policy according to tumor presentation. Journal of Clinical Oncology.

[ref-28] Shindle MK, Khanna AJ, McCarthy EF, O’Neill PJ, Sponseller PD (2002). Desmoid tumor of the spinal canal causing scoliosis and paralysis. Spine (Phila Pa 1976).

[ref-29] Skalli O, Gabbiani G, Babai F, Seemayer TA, Pizzolato G, Schurch W (1988). Intermediate filament proteins and actin isoforms as markers for soft tissue tumor differentiation and origin. II. Rhabdomyosarcomas. American Journal of Pathology.

[ref-30] Smith AJ, Lewis JJ, Merchant NB, Leung DH, Woodruff JM, Brennan MF (2000). Surgical management of intra-abdominal desmoid tumours. British Journal of Surgery.

[ref-31] Surowiak P, Murawa DV, Maciejczyk A, Pudelko M, Ciesla S, Breborowicz J, Murawa P, Zabel M, Dietel M, Lage H (2007). Occurence of stromal myofibroblasts in the invasive ductal breast cancer tissue is an unfavourable prognostic factor. Anticancer Research.

[ref-32] Tomita K, Kawahara N, Kobayashi T, Yoshida A, Murakami H, Akamaru T (2001). Surgical strategy for spinal metastases. Spine (Phila Pa 1976).

[ref-33] Zhang WG, Xu LB, Xiang YN, Duan CH (2018). Plexiform fibromyxoma of the small bowel: a case report. World Journal of Clinical Cases.

[ref-34] Zhao C, Han Z, Xiao H, Yang C, Zhao Y, Fan T, Sun Z, Liu T, Xiao J (2016). Surgical management of spinal liposarcoma: a case series of 7 patients and literature review. European Spine Journal.

[ref-35] Zhou Y, Chu X, Yi Y, Tong L, Dai Y (2017). Malignant solitary fibrous tumor in retroperitoneum: a case report and literature review. Medicine.

